# Supplementary material for: Excessive miR-25-3p maturation via N6-methyladenosine stimulated by cigarette smoke promotes pancreatic cancer progression
Source: Nat Commun. 2019 Apr 23;10:1858. doi: 10.1038/s41467-019-09712-x (PMC6478927; doi:10.1038/s41467-019-09712-x)
Supplement: Supplementary file 1 — Supplementary Information [file 41467_2019_9712_MOESM1_ESM.pdf]

Supplementary Information

Excessive miR-25-3p maturation via N^6-methyladenosine stimulated by cigarette smoke is vital in pancreatic cancer

Zhang *et al.*

Supplementary Figures

Supplementary Fig. 1: Association between the miR-25-3p level and survival in PDAC patients from the publicly available data.
Supplementary Fig. 2: Overexpression of miR-25-3p promotes malignant phenotypes of PDAC cells.
Supplementary Fig. 3: RNA levels of indicated genes in PDAC cells exposed to CSC or DMSO.
Supplementary Fig. 4: Efficiency of METTL3 and NKAP overexpression or knockdown in PDAC cells.
Supplementary Fig. 5: Potential transcription factors for METTL3 and correlation between their expression and METTL3 expression at mRNA levels.
Supplementary Fig. 6: Analysis of possible miR-25-3p binding sites in PHLPP2 3′untranslated region (3′UTR).
Supplementary Fig. 7: MiR-25-3p activates AKT-p70S6K signaling.
Supplementary Fig. 8: Unprocessed gel blots underlying Figs. 2 and 3.
Supplementary Fig. 9: Unprocessed gel blots underlying Figs. 4, 6d and f.
Supplementary Fig. 10: Unprocessed gel blots underlying Figs. 6g, h and 7.
Supplementary Fig. 11: Unprocessed gel blots underlying Supplementary Figs. 3 and 7.

Supplementary Tables

Supplementary Table 1: Baseline demographic and clinical characteristics of individuals with pancreatic ductal adenocarcinoma (PDAC) in this study.
Supplementary Table 2: Associations between miR-25-3p level and clinical characteristics of individuals with pancreatic ductal adenocarcinoma (PDAC) in this study.
Supplementary Table 3: Characteristics of subjects for serum miR-25-3p analysis in this study.
Supplementary Table 4: [m^6A]Pri-miR-25 and DGCR8 interacting nuclear proteins identified by RNA pulldown or immunoprecipitation assays followed by mass spectrometry analysis.
Supplementary Table 5: Primers used for quantitative real time-PCR and other assays in this study.
Supplementary Table 6: Sequences of siRNAs, primers and probes used in this study.

Supplementary Fig. 1

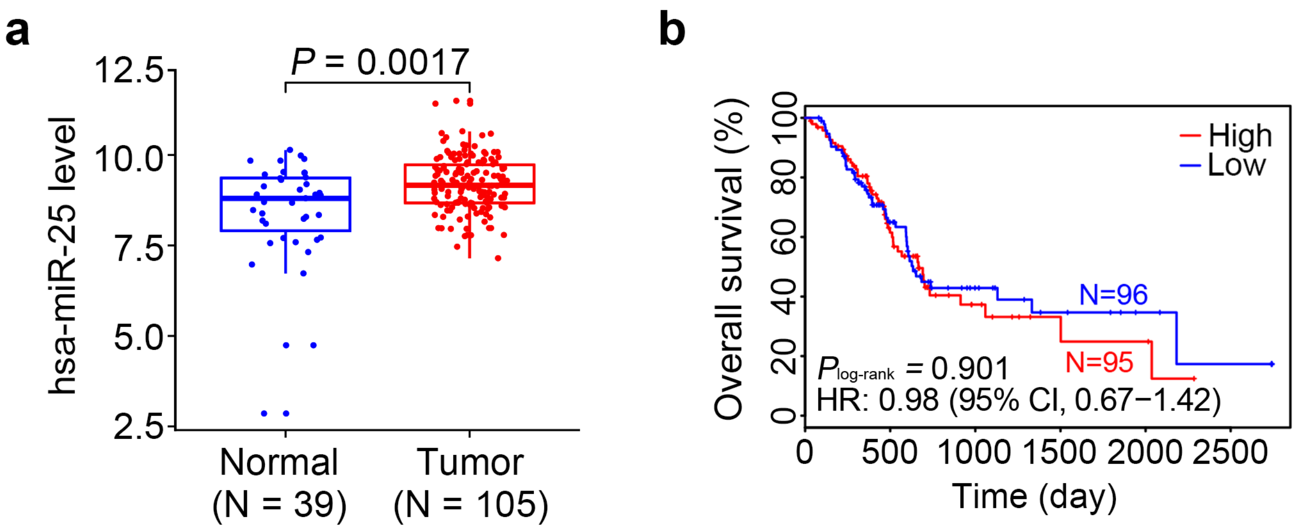

Supplementary Fig. 1. Association between the miR-25-3p level and survival in PDAC patients from the publicly available data. a Higher level of miR-25-3p was detected in PDAC tissue compared with normal tissue revealed by integrate analysis of microRNA array data from Gene Expression Omnibus (GSE24279, GSE25820 and GSE41369). *P* value was analysed by unpaired Student *t* test. **b** Kaplan-Meier plots showed non-significant correlation between miR-25-3p level and overall survival rate of patients with PDAC in TCGA databse. Analysis using TCGA data was performed by PROGmiR (see URLs).

Supplementary Fig. 2

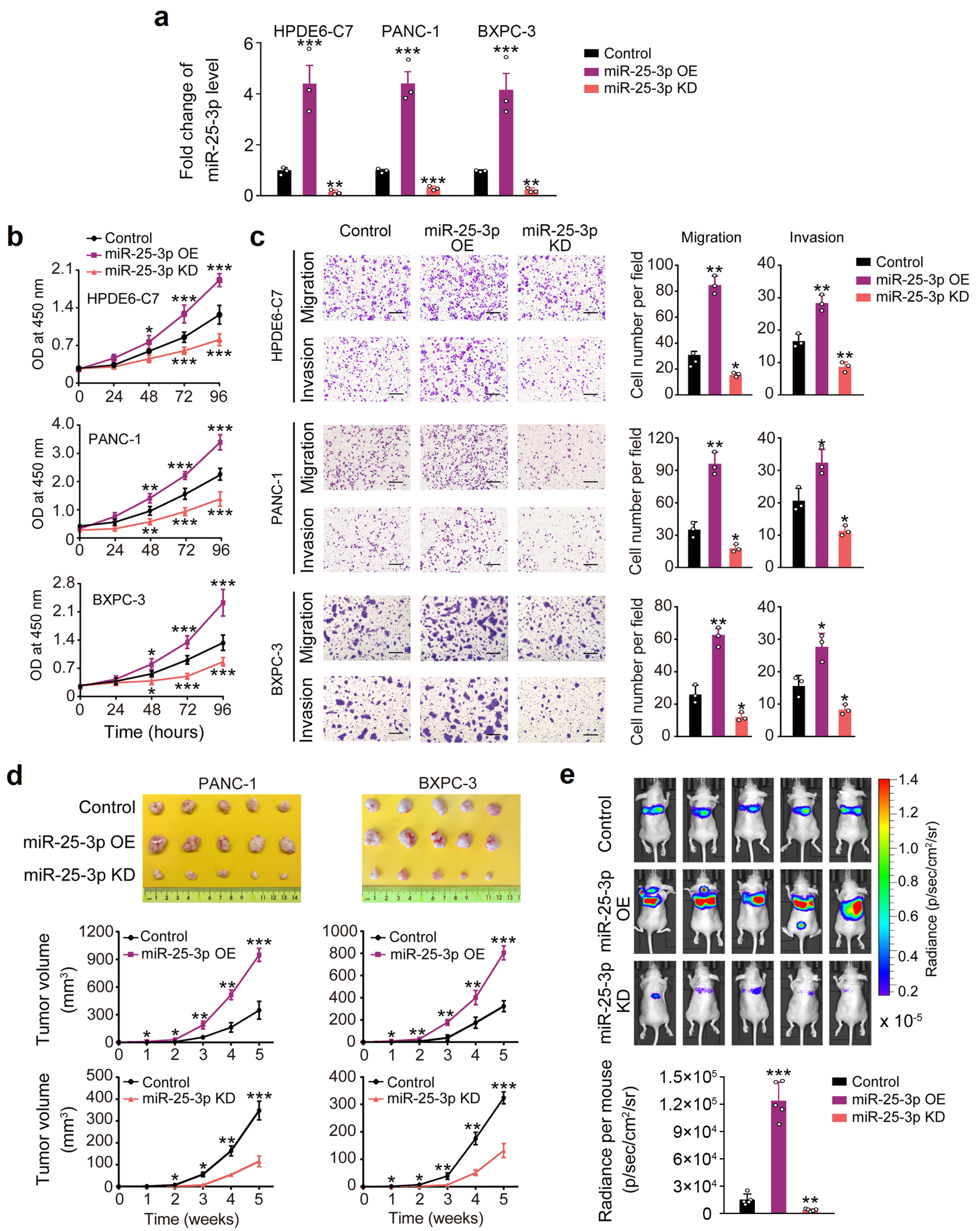

Supplementary Fig. 2. Overexpression of miR-25-3p promotes malignant phenotypes of PDAC cells. a Overexpression or knockdown of miR-25-3p in HPDE6-C7 and PDAC cells. Results represent means ± S.D. from 3 independent qRT-PCR determinations. **b** Effects of miR-25-3p on in vitro proliferation of HPDE6-C7 and PDAC cells. Results are means ± S.D. from 3 experiments and each had 6 replicates. **c** Effects of miR-25-3p on migration and invasion of HPDE6-C7 and PDAC cells in vitro. *Left panels* are representative pictures of transwell assays showing different abilities of cell migration and invasion; *right panels* show quantitative statistics of migration and invasion abilities. Data are means ± S.D. from 3 random fields. Scale bars, 200 μm. **d** Effects of miR-25-3p on growth of xenograft tumors derived from PDAC cells *in vivo* in nude mice. *Upper panels* show subcutaneous xenografts obtained at the end of experiments and *lower panels* are the curves of xenograft growth. Data represent means ± S.D. from 5 mice of each group. **e** Effects of miR-25-3p on lung localization of PDAC cells in nude mice ($N = 5$) with injection of cells via tail-vein. *Upper panels* show bioluminescence imaging at day 42 after injection of cells, and *lower panel* shows quantitative fluorescent intensities. Data are means ± S.D. All statistic examinations in this figure are Student *t* test. *$P < 0.05$, **$P < 0.01$ and ***$P < 0.001$ as compared with the corresponding control.

Supplementary Fig. 3

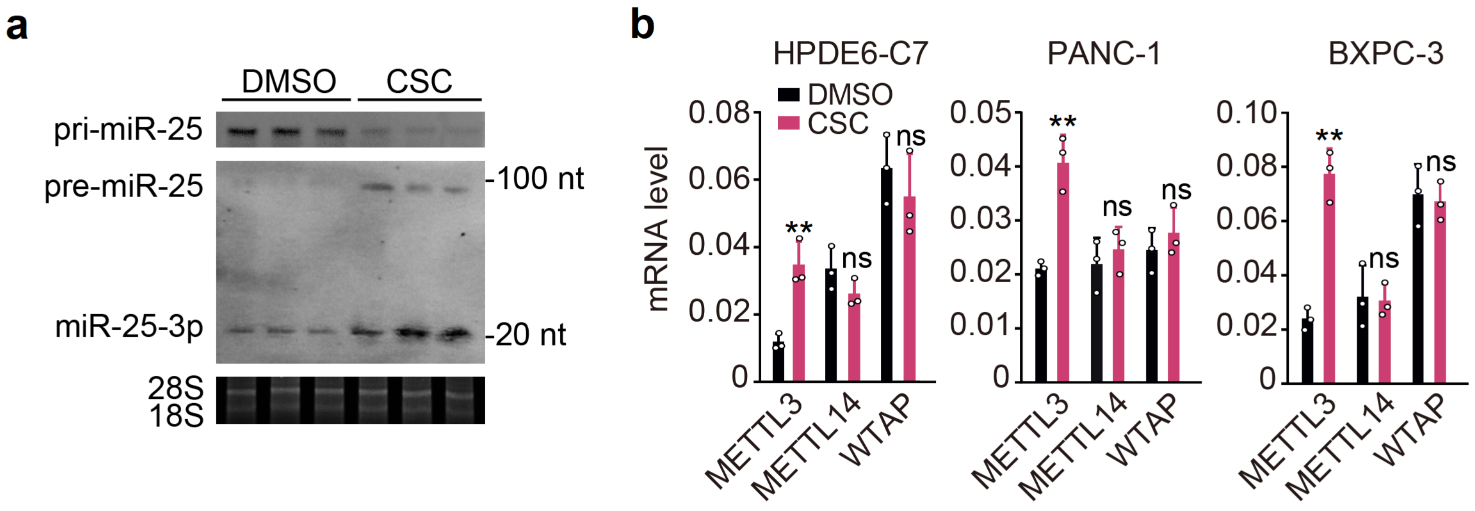

Supplementary Fig. 3. RNA levels of indicated genes in PDAC cells exposed to CSC or DMSO. **a** Northern blot showing the levels of pri-miR-25, pre-miR-25 and mature miR-25-3p in PANC-1 cells treated with CSC (100 μg/ml) or DMSO as solvent control. 28S and 18S rRNAs were served as loading controls. **b** RNA levels of 3 indicated genes in HPDE6-C7 and PDAC cells exposed to CSC (100 μg/ml) or solvent. Results are means ± S.D. from 3 measurements by qRT-PCR normalized to ACTIN. ***P* < 0.01 by Student *t* test compared with control. ns, not significant.

Supplementary Fig. 4

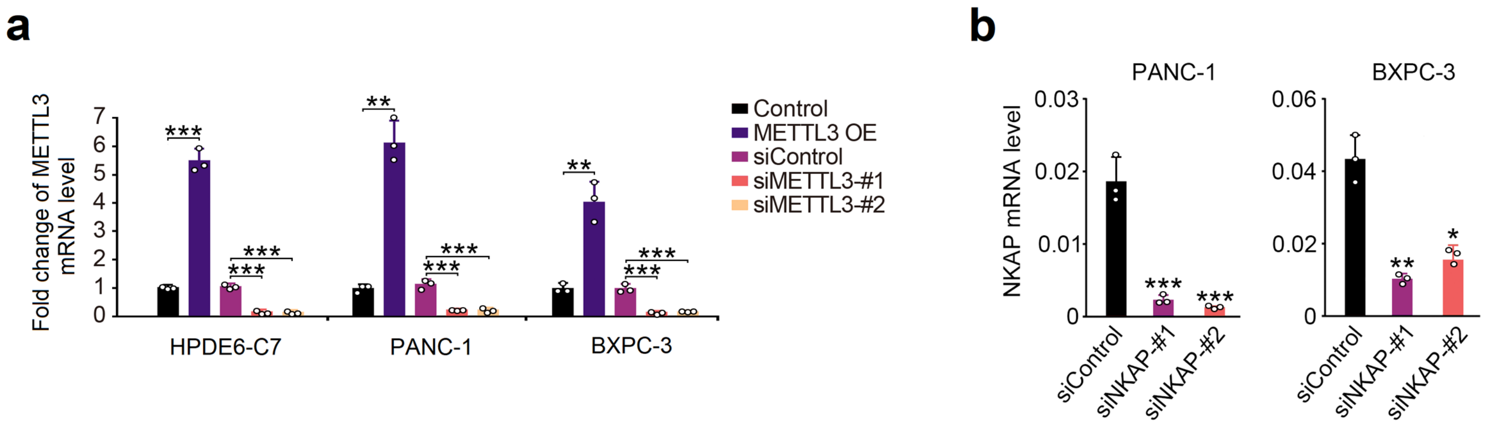

Supplementary Fig. 4. Efficiency of METTL3 and NKAP overexpression or knockdown in PDAC cells. a METTL3 RNA levels in HPDE6-C7, PANC-1 and BXPC-3 cells transfected with empty vector or vector containing METTL3 or METTL3 siRNA. **b** NKAP RNA levels in PDAC cells transfected with NKAP siRNA or control vector. Results are means ± S.D. from 3 measurements by qRT-PCR normalized to ACTIN. *$P < 0.05$, **$P < 0.01$ and ***$P < 0.001$ by Student *t* test compared with each control.

Supplementary Fig. 5

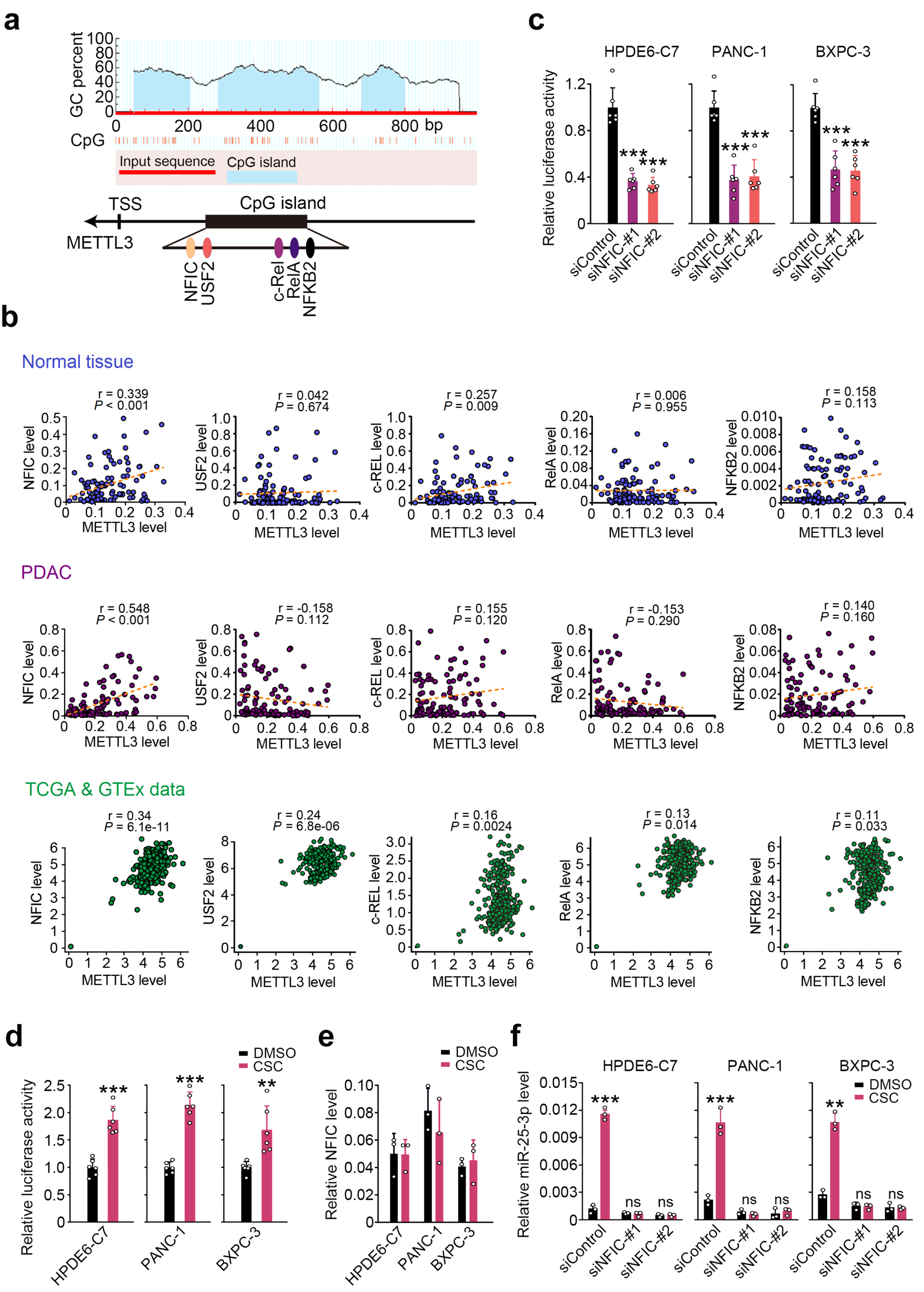

Supplementary Fig. 5. Potential transcription factors for *METTL3* and correlation between their expression and *METTL3* expression at mRNA levels. a *In silico* analysis with the JARSPER online database suggested transcription factors binding to the CpG island of *METTL3* promoter. **b** Pearson correlations between expressions of suggested transcription factors and *METTL3* at mRNA levels in our samples of non-tumor pancreatic tissues (N = 102, *upper panel*) or PDAC (N = 102, *middle panel*) and in PDAC samples of integrated TCGA and Genotype-Tissues Expression (GTEx) data (Normal, N = 171; Tumor, N = 179, *lower panel*). Integrated analysis of public data was performed using GEPIA (see URLs). **c** Effects of NFIC knockdown on transcriptional activity of the *METTL3* promoter as detected by luciferase reporter assays. **d** Effects of CSC (100 μg/ml) or DMSO treatment on transcriptional activity of the *METTL3* promoter as detected by luciferase reporter assays. **e** *NFIC* mRNA levels in HPDE6-C7 and PDAC cells exposed to CSC (100 μg/ml) or DMSO. **f** *NFIC* knockdown significantly affected the expression of miR-25-3p in HPDE6-C7, PANC-1 and BXPC-3 cells treated with CSC (100 μg/ml). Data of c−f are means ± S.D. All statistic examinations in this figure are Student *t* test. **P < 0.01 and ***P < 0.001 as compared with the corresponding control.

Supplementary Fig. 6

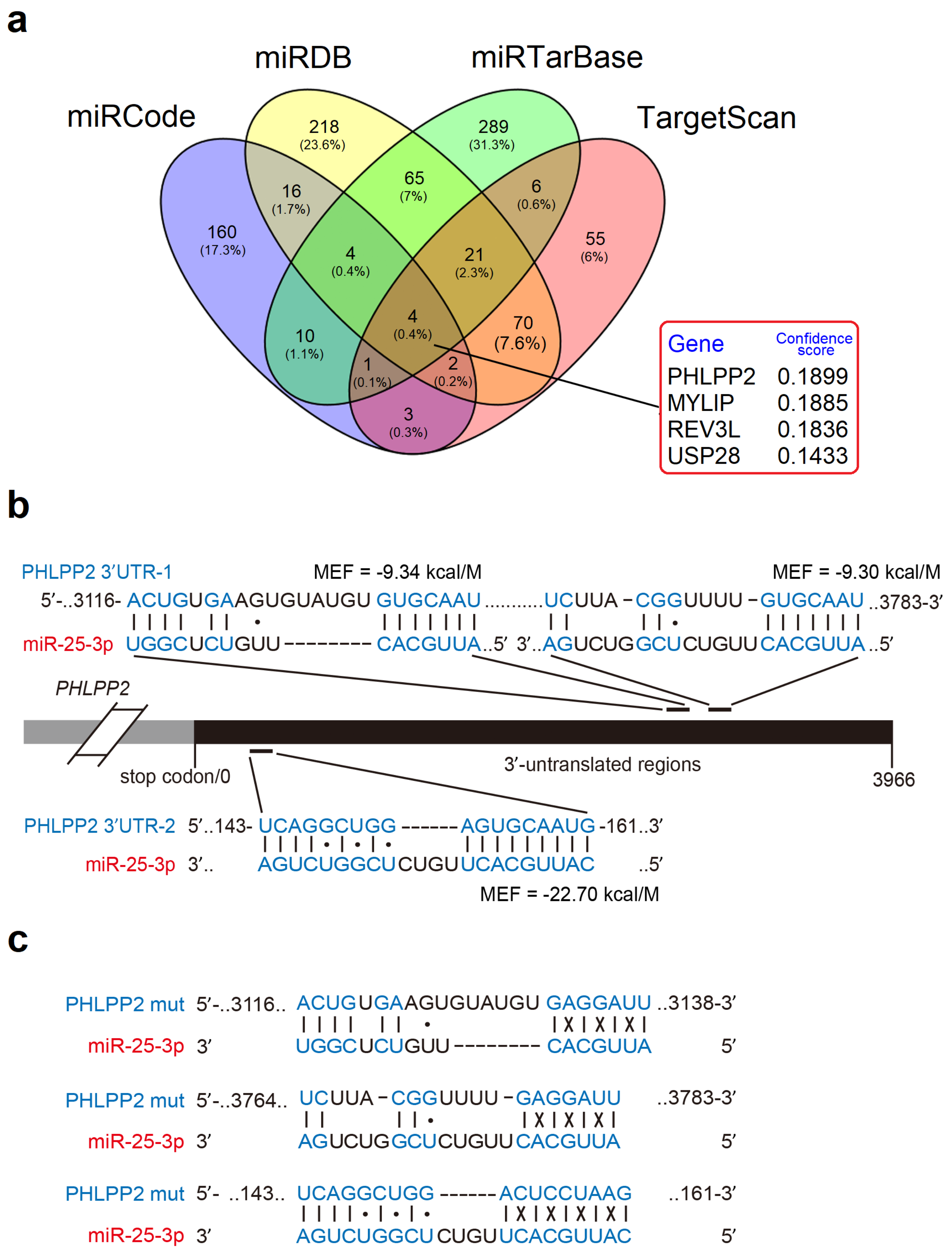

Supplementary Fig. 6. Analysis of possible miR-25-3p binding sites in *PHLPP2* 3′untranslated region (3′UTR). **a** Venn diagram showing potential target genes of miR-25-3p in 4 databases. Among the 4 genes overlapped in the 4 databases, *PHLPP2* ranged the highest confidence as a target of miR-25-3p. Confidence score measured by an algorithm from mirDIP combining the binding energy, evolutionary conservation, ranks and associated precisions. Higher scores indicate more confidence of the prediction. **b** Shown are the three possible miR-25-3p binding sites within the *PHLPP2* 3′UTR. **c** Shown are the site-specific mutations in the seed sequences of the *PHLPP2* 3′UTR where miR-25-3p might target.

Supplementary Fig. 7

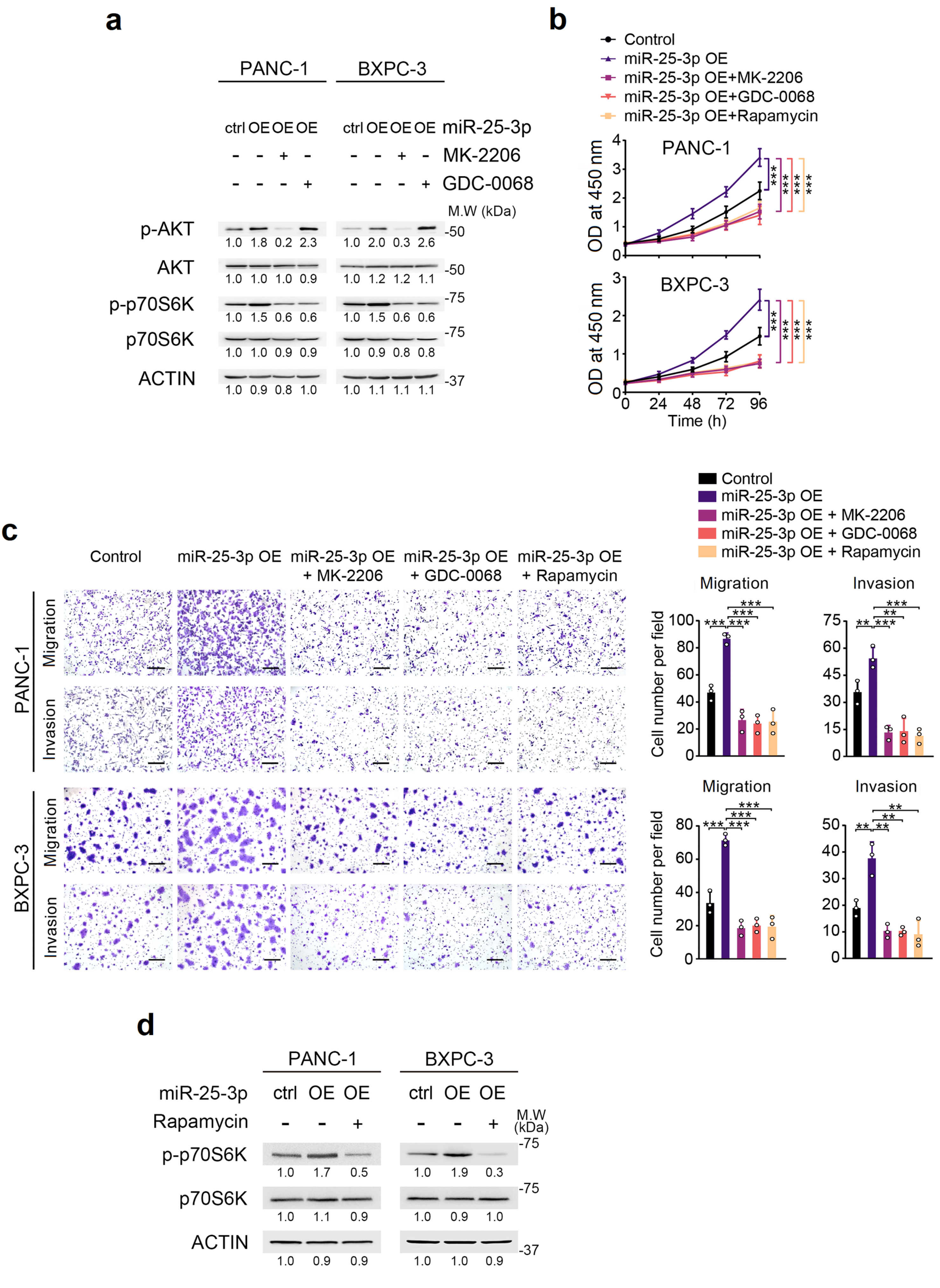

Supplementary Fig. 7. MiR-25-3p activates AKT-p70S6K signaling. a The AKT inhibitors MK-2206 (10 μM) and GDC-0068 (10 μM) substantially abolished the effects of miR-25-3p, suggesting the microRNA as an upstream activator of AKT signaling via inhibiting PHLPP2 production. **b** AKT inhibitors (MK-2206 and GDC-0068) or p70S6K inhibitor (Rapamycin, 100 nM) significantly reversed the effects of miR-25-3p overexpression on PDAC cell proliferation. Results represent mean ± S.D. from 3 independent experiments. **c** AKT inhibitors or p70S6K inhibitor significantly reversed the effects of miR-25-3p overexpression on PDAC cell migration and invasion. *Upper panel* are representative images of transwell assays and *lower panel* are quantitative statistics. Scale bars, 200 μm. Results are mean ± S.D. from 3 random fields. **d** The p70S6K inhibitor rapamycin substantially abolished miR-25-3p-enhanced p70S6K activation. $^{**}P < 0.01$ and $^{***}P < 0.001$ by Student *t* test compared with each control.

Supplementary Fig. 8

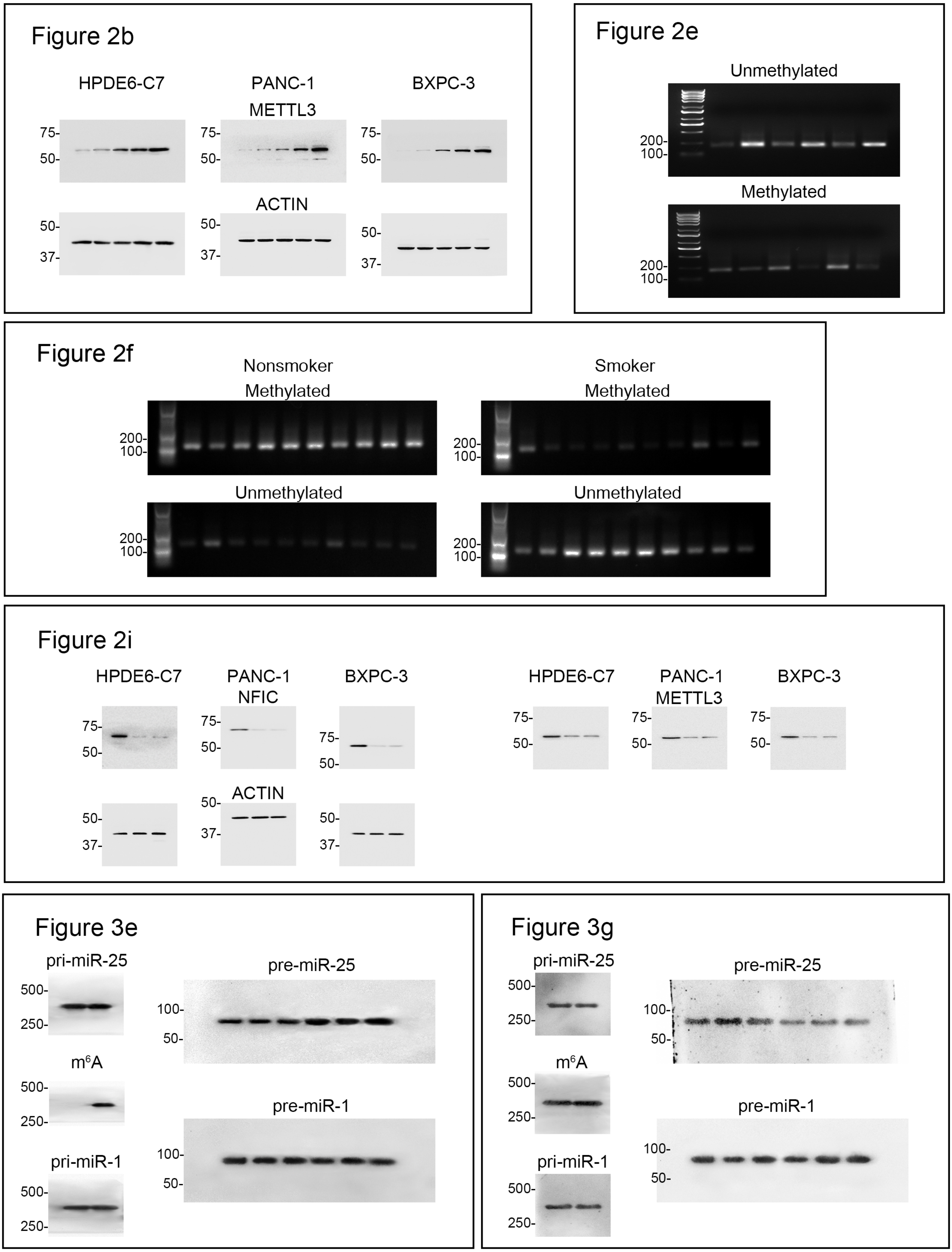

Supplementary Fig. 8. Unprocessed gel blots. Of note, for some immunoblotting assays membranes were cut into several pieces to incubate with different antibodies, and therefore the raw images of these membranes are of small size.

Supplementary Fig. 9

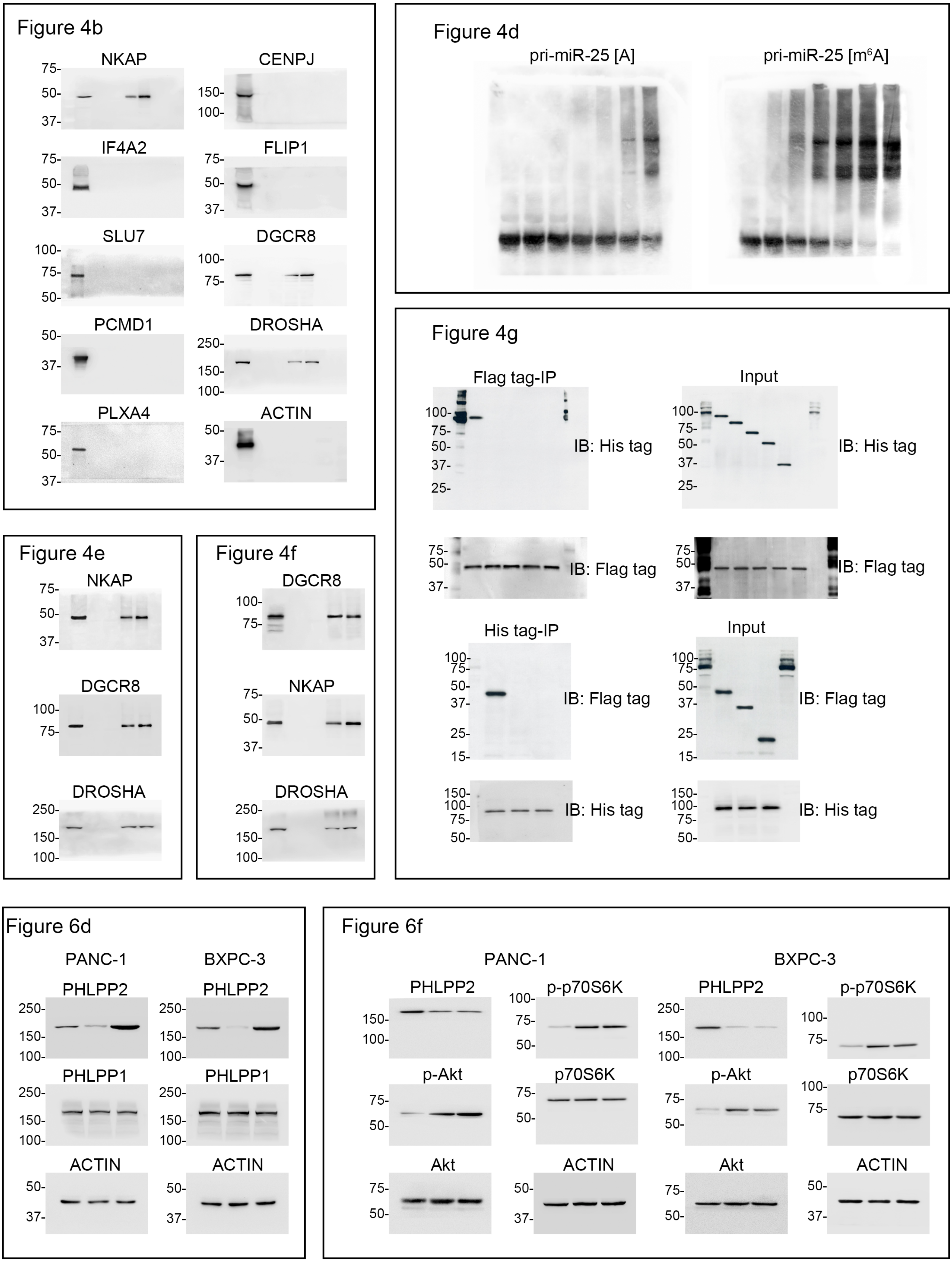

Supplementary Fig. 9. Unprocessed gel blots. (Continued)

Supplementary Fig. 10

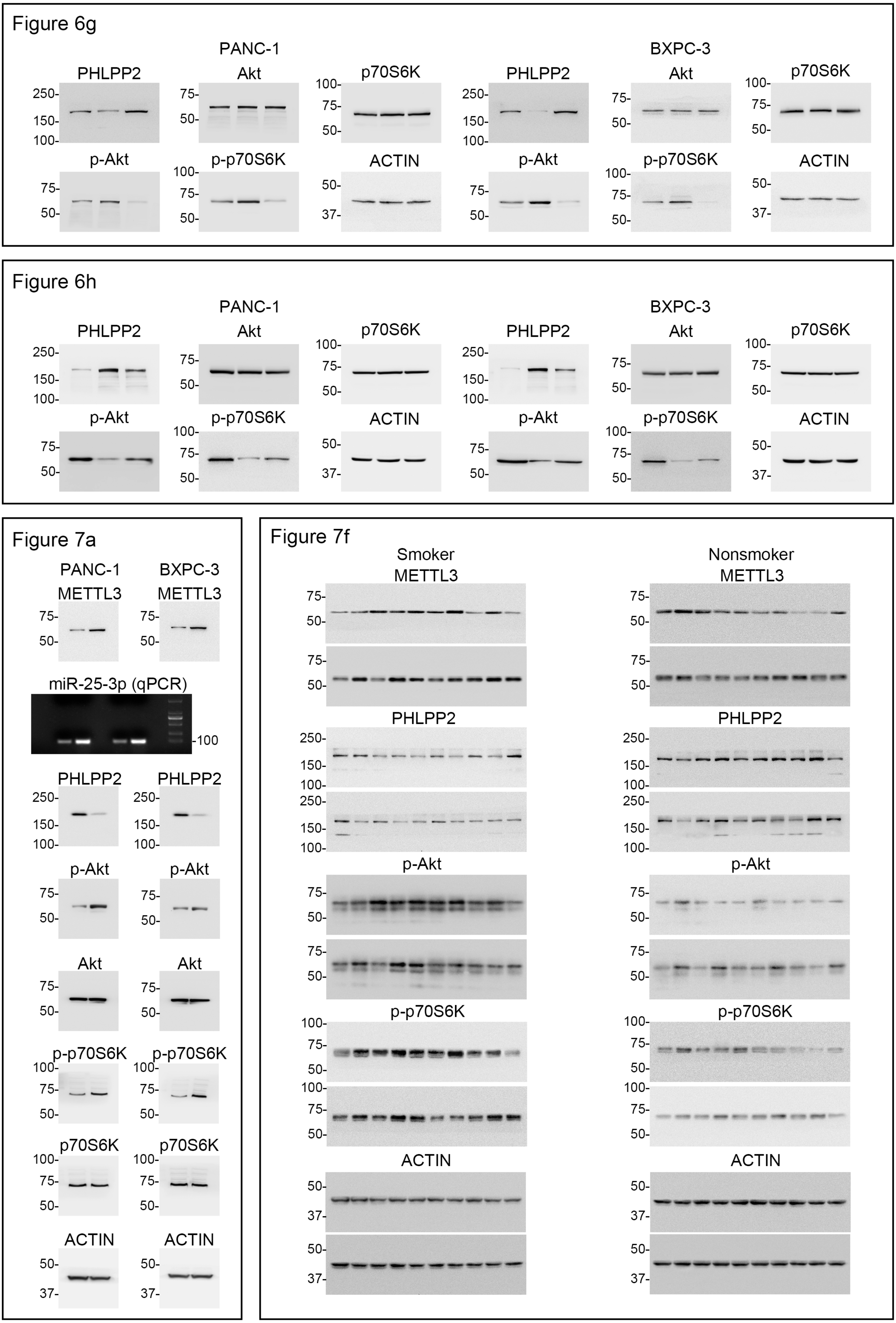

Supplementary Fig. 10. Unprocessed gel blots. (Continued)

Supplementary Fig. 11

Supplementary Figure 3

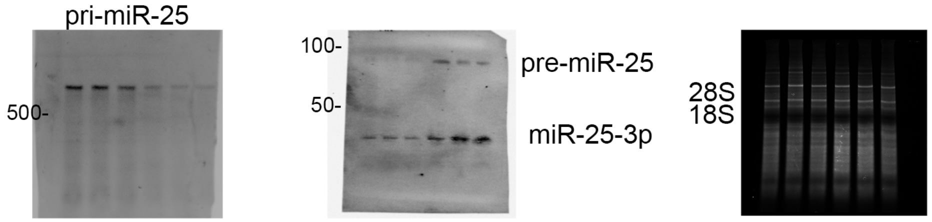

Supplementary Figure 7a

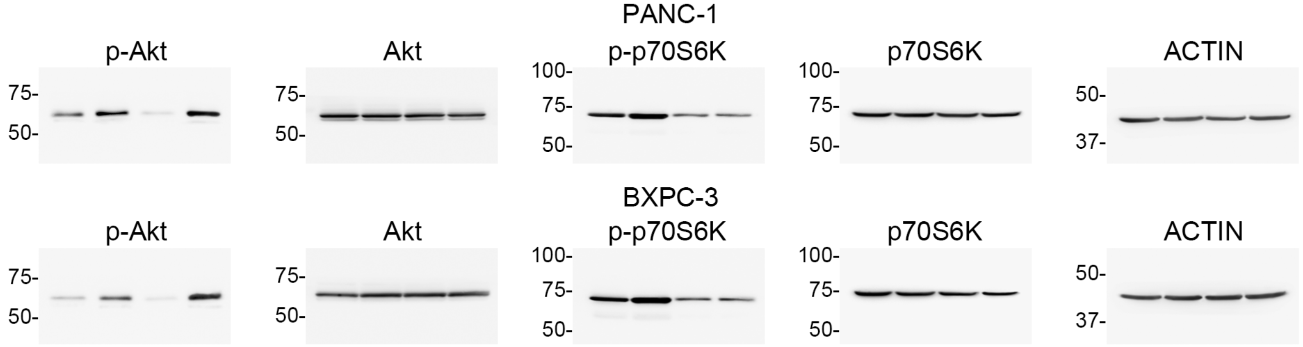

Supplementary Figure 7d

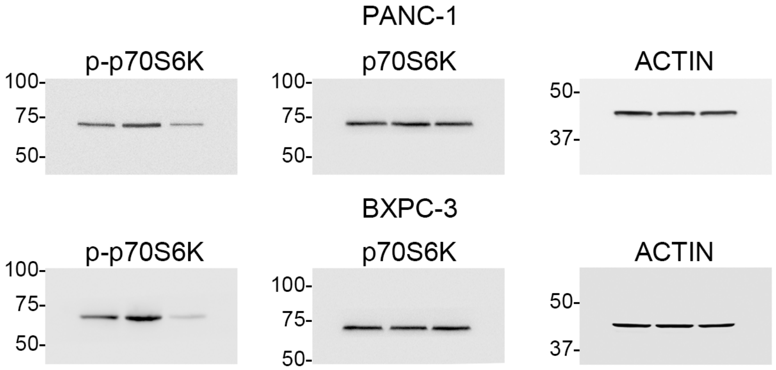

Supplementary Fig. 11. Unprocessed gel blots. (Continued)

Supplementary Table 1. Baseline demographic and clinical characteristics of individuals with pancreatic ductal adenocarcinoma (PDAC) in this study.

	Cohort 1 (Guangzhou)			Cohort 2 (Beijing)			Pooled sample		
	Alive (*N* = 38)	Deceased (*N* = 64)	*P*	Alive (*N* = 17)	Deceased (*N* = 56)	*P*	Alive (*N* = 55)	Deceased (*N* = 120)	*P*
Age, mean (S.E.M.[a])	59.3 (2.1)	60.2 (1.3)	0.567	59.2(2.1)	63.1 (1.0)	0.186	59.2 (1.5)	61.5 (1.0)	0.193
Sex, *N* (%)			0.506			0.785			0.433
Male	20 (52.6)	38 (59.4)		10 (58.8)	35 (62.5)		30 (54.5)	73 (60.8)	
Female	18 (47.4)	26 (40.6)		7 (41.2)	21 (37.5)		25 (45.5)	47 (39.2)	
Differentiation, *N* (%)			0.616			0.254			0.532
Well	7 (18.4)	17 (26.5)		4 (23.6)	6 (10.7)		11 (20.0)	23 (19.2)	
Moderate	24 (63.2)	35 (54.7)		10 (58.8)	31 (55.4)		34 (61.8)	66 (55.0)	
Poor	7 (18.4)	12 (18.8)		3 (17.6)	19 (33.9)		10 (18.2)	31 (25.8)	
Lymph node metastasis, *N* (%)			0.028			0.443			0.196
Positive	14 (36.8)	38 (59.4)		10 (58.8)	27 (48.2)		24 (43.6)	65 (54.2)	
Negative	24 (63.2)	26 (40.6)		7 (41.2)	29 (51.8)		31 (56.4)	55 (45.8)	
Vascular invasion, *N* (%)			0.156			0.349			0.045
Yes	7 (18.4)	20 (31.2)		6 (35.3)	27 (48.2)		13 (23.6)	47 (39.2)	
No	31 (81.6)	44 (68.8)		11 (64.7)	29 (51.8)		42 (76.4)	73 (60.8)	
Neural invasion, *N* (%)			0.254			0.603			0.852
Yes	14 (36.8)	31 (48.4)		4 (23.5)	10 (17.9)		18 (32.7)	41 (34.2)	
No	24 (63.2)	33 (51.6)		13 (76.5)	46 (82.1)		37 (67.3)	79 (65.8)	
TNM stage[b], *N* (%)			0.011			0.220			0.529
I	3 (7.9)	4 (6.3)		4 (23.5)	9 (16.0)		7 (12.7)	13 (10.8)	
II	23 (60.5)	55 (85.9)		11 (64.7)	27 (48.2)		34 (61.8)	82 (68.4)	
III	2 (5.3)	2 (3.1)		2 (11.8)	10 (17.9)		4 (7.3)	12 (10.0)	
IV	10 (26.3)	3 (4.7)		0 (0.0)	10 (17.9)		10 (18.2)	13 (10.8)	
Smoking status, *N* (%)			0.802			0.832			0.752
Ever	17 (44.7)	27 (42.2)		5 (29.4)	18 (32.1)		22 (40.0)	45 (37.5)	
Never	21 (55.3)	37 (57.8)		12 (70.6)	38 (67.9)		33 (60.0)	75 (62.5)	
Drinking status, *N* (%)			0.206			0.974			0.267
Ever	5 (13.2)	15 (23.4)		5 (29.4)	16 (28.6)		10 (18.2)	31 (25.8)	
Never	33 (86.8)	49 (76.6)		12 (70.6)	40 (71.4)		45 (81.8)	89 (74.2)	
Treatment, *N* (%)			0.879			0.964			0.845
Surgery only	19 (50.0)	31 (48.4)		8 (47.1)	26 (46.4)		27 (49.1)	57 (47.5)	
Surgery + Chemotherapy	19 (50.0)	33 (51.6)		9 (52.9)	30 (53.6)		28 (50.9)	63 (52.5)	

[a]S.E.M., standard error of mean.

[b]Tumor TNM staging were reviewed by at least 3 pathologists and defined according to the American Joint Committee on Cancer (AJCC) 7th edition.

Supplementary Table 2. Associations between miR-25-3p level and clinical characteristics of individuals with pancreatic ductal adenocarcinoma (PDAC) in this study.

	Cohort 1 (Guangzhou)[a]			Cohort 2 (Beijing)[a]			Pooled sample[a]		
	Low miR-25-3p (*N* = 51)	High miR-25-3p (*N* = 51)	*P*	Low miR-25-3p (*N* = 36)	High miR-25-3p (*N* = 37)	*P*	Low miR-25-3p (*N* = 87)	High miR-25-3p (*N* = 88)	*P*
Age, mean (S.E.M.)	60.6 (1.7)	59.2 (1.4)	0.551	62.6 (1.8)	61.7 (1.8)	0.704	61.4 (1.1)	60.2 (1.2)	0.488
Sex, *N* (%)			0.689			0.926			0.807
Male	30 (58.8)	28 (54.9)		22 (61.1)	23 (62.2)		52 (59.8)	51 (58.0)	
Female	21 (41.2)	23 (45.1)		14 (38.9)	14 (37.8)		35 (40.2)	37 (42.0)	
Differentiation, *N* (%)			0.587			0.995			0.782
Well	11 (21.6)	13 (25.5)		5 (13.8)	5 (13.5)		16 (18.4)	18 (20.5)	
Moderate	32 (62.7)	27 (52.9)		20 (55.6)	21 (56.8)		52 (59.8)	48 (54.5)	
Poor	8 (15.7)	11 (21.6)		11 (30.6)	11 (29.7)		19 (21.8)	22 (25.0)	
Lymph node metastasis, *N* (%)			0.113			0.908			0.199
Positive	22 (43.1)	30 (58.8)		18 (50.0)	19 (51.4)		40 (46.0)	49 (55.7)	
Negative	29 (56.9)	21 (41.2)		18 (50.0)	18 (48.6)		47 (54.0)	39 (44.3)	
Vascular invasion, *N* (%)			0.822			0.733			0.956
Yes	13 (25.5)	14 (27.5)		17 (47.2)	16 (43.2)		30 (34.5)	30 (34.1)	
No	38 (74.5)	37 (72.5)		19 (52.8)	21 (56.8)		57 (65.5)	58 (65.9)	
Neural invasion, *N* (%)			0.550			0.955			0.670
Yes	21 (41.2)	24 (47.1)		7 (19.4)	7 (18.9)		28 (32.2)	31 (35.2)	
No	30 (58.8)	27 (52.9)		29 (80.6)	30 (81.1)		59 (67.8)	57 (64.8)	
TNM stage[b], *N* (%)			0.501			0.703			0.998
I	2 (3.9)	5 (9.8)		8 (22.2)	5 (13.5)		10 (11.5)	10 (11.4)	
II	39 (76.5)	39 (76.4)		19 (52.8)	19 (51.4)		58 (66.7)	58 (65.9)	
III	3 (5.9)	1 (2.0)		5 (13.9)	7 (18.9)		8 (9.2)	8 (9.1)	
IV	7 (13.7)	6 (11.8)		4 (11.1)	6 (16.2)		11 (12.6)	12 (13.6)	
Smoking status, *N* (%)			0.016			0.238			0.010
Ever	16 (31.4)	28 (54.9)		9 (25.0)	14 (37.8)		25 (28.7)	42 (47.7)	
Never	35 (68.6)	23 (45.1)		27(75.0)	23 (62.2)		62 (71.3)	46 (52.3)	
Drinking status, *N* (%)			0.618			0.483			0.891
Ever	11 (21.6)	9 (17.6)		9 (25.0)	12 (32.4)		20 (23.0)	21 (23.9)	
Never	40 (78.4)	42 (82.4)		27 (75.0)	25 (67.6)		67 (77.0)	67 (76.1)	

[a]Low or high miR-25-3p level is defined as <median or ≥median.

[b]TNM staging were reviewed by at least 3 pathologists and defined according to the American Joint Committee on Cancer (AJCC) 7th edition.

Supplementary Table 3. Characteristics of subjects for serum miR-25-3p analysis in this study.

Sample ID	Sex	Age	Smoking status	Years smoked	Cigarettes per day
S1	Male	54	Smoker	36	20
S2	Male	55	Smoker	35	20
S3	Male	41	Smoker	28	20
S4	Male	58	Smoker	18	20
S5	Male	55	Smoker	35	10
S6	Male	43	Smoker	25	20
S7	Male	40	Smoker	20	10
S8	Male	52	Smoker	22	10
S9	Male	43	Smoker	18	20
S10	Male	51	Smoker	31	20
S11	Male	49	Smoker	19	20
S12	Male	38	Smoker	18	20
S13	Male	30	Smoker	8	10
S14	Male	29	Smoker	9	20
S15	Male	32	Smoker	3	15
S16	Male	37	Smoker	14	15
S17	Female	42	Smoker	20	20
S18	Female	70	Smoker	36	20
S19	Female	68	Smoker	38	20
S20	Female	45	Smoker	10	10
S21	Female	61	Smoker	9	20
S22	Female	69	Smoker	39	10
NS1	Male	26	Nonsmoker	–	–
NS2	Male	35	Nonsmoker	–	–
NS3	Male	25	Nonsmoker	–	–
NS4	Male	51	Nonsmoker	–	–
NS5	Male	38	Nonsmoker	–	–
NS6	Male	39	Nonsmoker	–	–
NS7	Male	29	Nonsmoker	–	–
NS8	Male	34	Nonsmoker	–	–
NS9	Male	38	Nonsmoker	–	–
NS10	Male	84	Nonsmoker	–	–
NS11	Male	77	Nonsmoker	–	–
NS12	Male	70	Nonsmoker	–	–
NS13	Male	69	Nonsmoker	–	–
NS14	Male	56	Nonsmoker	–	–
NS15	Male	49	Nonsmoker	–	–
NS16	Male	42	Nonsmoker	–	–
NS17	Female	54	Nonsmoker	–	–
NS18	Female	32	Nonsmoker	–	–
NS19	Female	36	Nonsmoker	–	–
NS20	Female	37	Nonsmoker	–	–
NS21	Female	34	Nonsmoker	–	–
NS22	Female	41	Nonsmoker	–	–
NS23	Female	41	Nonsmoker	–	–

Supplementary Table 4. [m^{6}A]Pri-miR-25 and DGCR8 interacting nuclear proteins identified by RNA pulldown or immunoprecipitation assays followed by mass spectrometry analysis.

Protein name (symbol)	Gene name	NCBI-accession
NF-kappa-B-activating protein (NKAP)	*NKAP*	Q8N5F7
Pre-mRNA-splicing factor SLU7 (SLU7)	*SLU7*	O95391
Protein-L-isoaspartate *O*-methyltransferase domain-containing protein 1 (PCMTD1)	*PCMTD1*	Q96MG8
Eukaryotic initiation factor 4A-II (IF4A2)	*IF4A2*	Q14240
Ribonuclease 3 (RNC)	*DROSHA*	Q9NRR4
Microprocessor complex subunit DGCR8 (DGCR8)	*DGCR8*	Q8WYQ5
Plexin-A4 (PLXA4)	*PLXNA4*	Q9HCM2
Centromere protein J (CENPJ)	*CENPJ*	Q9HC77
Filamin-A-interacting protein 1 (FLIP1)	*FILIP1*	Q7Z7B0

Supplementary Table 5. Primers used for quantitative real time-PCR and other assays in this study.

Gene Symbol	Forward primer (5'→3')	Reverse primer (5'→3')
NFIC	GCTGGACACGACCGACTTC	CCGGGACACTTGGATGAGC
USF2	CCCGGACACACCCTTACTCT	GCTCCACTTCGTTGTGCTG
c-Rel	CAACCGAACATACCCTTCTATCC	TCTGCTTCATAGTAGCCGTCT
RelA	CCCACGAGCTTGTAGGAAAGG	GGATTCCCAGGTTCTGGAAAC
NFKB2	AGAGGCTTCCGATTTCGATATGG	GGATAGGTCTTTCGGCCCTTC
DGCR8	AGCGTGAGCTTTACCGAGAG	CTACCCCGTCACCAACACTC
DROSHA	CCCTCCGGGCTATTCTCAC	TGGTCATCATAGTGTTTCAGCCT
NKAP	TGAAGATAGCGACAGTGACTCT	CAGCCTTTGTTCGATCCTTCC
METTL3	TTGTCTCCAACCTTCCGTAGT	CCAGATCAGAGAGGTGGTGTAG
METTL14	GAGTGTGTTTACGAAAATGGGGT	CCGTCTGTGCTACGCTTCA
WTAP	CTTCCCAAGAAGGTTCGATTGA	TCAGACTCTCTTAGGCCAGTTAC
PHLPP2	TGGAACCTACTGAACGACCTC	ATCCAAACGATCCATGTGGCA
hsa-pri-miR-1	TATGTATCTCAGGCCGGGACC	GGACACGACCGTCCACCA
hsa-pri-miR-25	TAAGAAACTCCGGGACTGGCCAGTG	CACTGTCAGACCGAGACAAGTGCAA
β-ACTIN	CAGGGCGTGATGGTGGGCATG	GTAGAAGGTGTGGTGCCAGATT
Synthesized *Caenorhabditis* elegans miRNA		
cel-miR-39-oligo	UCACCGGGUGUAAAUCAGCUUG	
cel-miR-54-oligo	UACCCGUAAUCUUCAUAAUCCGAG	
Reverse transcription primers		
hsa-miR-25-3p	GTCGTATCCAGTGCGTGTCGTGGAGTCGGCAATTGCACTGGATACGACTCAGACCG	
hsa-miR-1-1-3p	GTCGTATCCAGTGCGTGTCGTGGAGTCGGCAATTGCACTGGATACGACATACATAC	
hsa-miR-145-5p	GTCGTATCCAGTGCGTGTCGTGGAGTCGGCAATTGCACTGGATACGACAGGGATTC	
hsa-miR-4763-3p	GTCGTATCCAGTGCGTGTCGTGGAGTCGGCAATTGCACTGGATACGACCCCGCCCA	
hsa-miR-630	GTCGTATCCAGTGCGTGTCGTGGAGTCGGCAATTGCACTGGATACGACACCTTCCCT	
hsa-miR-1229-5p	GTCGTATCCAGTGCGTGTCGTGGAGTCGGCAATTGCACTGGATACGACCGCTCTCC	
hsa-miR-5703	GTCGTATCCAGTGCGTGTCGTGGAGTCGGCAATTGCACTGGATACGACACCTTCCCG	
hsa-miR-6727-5p	GTCGTATCCAGTGCGTGTCGTGGAGTCGGCAATTGCACTGGATACGACCGCTCCCA	
hsa-miR-328-5p	GTCGTATCCAGTGCGTGTCGTGGAGTCGGCAATTGCACTGGATACGACCCCTGAGC	
hsa-miR-5787	GTCGTATCCAGTGCGTGTCGTGGAGTCGGCAATTGCACTGGATACGACACCTCCCC	
hsa-miR-34a-3p	GTCGTATCCAGTGCGTGTCGTGGAGTCGGCAATTGCACTGGATACGACAGGGCAGT	
hsa-miR-4516	GTCGTATCCAGTGCGTGTCGTGGAGTCGGCAATTGCACTGGATACGACGCCCCGAC	
hsa-miR-4459	GTCGTATCCAGTGCGTGTCGTGGAGTCGGCAATTGCACTGGATACGACCTCCACCT	
hsa-miR-6090	GTCGTATCCAGTGCGTGTCGTGGAGTCGGCAATTGCACTGGATACGACGCCCCGCC	
hsa-miR-7107-5p	GTCGTATCCAGTGCGTGTCGTGGAGTCGGCAATTGCACTGGATACGACCCCTTCCT	
hsa-miR-6068	GTCGTATCCAGTGCGTGTCGTGGAGTCGGCAATTGCACTGGATACGACCCACCGCC	
hsa-miR-6869-5p	GTCGTATCCAGTGCGTGTCGTGGAGTCGGCAATTGCACTGGATACGACGCCGCCGC	
hsa-miR-33a-5p	GTCGTATCCAGTGCGTGTCGTGGAGTCGGCAATTGCACTGGATACGACTGCAATGC	
hsa-miR-582-3p	GTCGTATCCAGTGCGTGTCGTGGAGTCGGCAATTGCACTGGATACGACGGTTCAGT	
hsa-miR-30c-2-3p	GTCGTATCCAGTGCGTGTCGTGGAGTCGGCAATTGCACTGGATACGACAGAGTAAA	
hsa-miR-181d-5p	GTCGTATCCAGTGCGTGTCGTGGAGTCGGCAATTGCACTGGATACGACACCCACCG	
hsa-miR-186-5p	GTCGTATCCAGTGCGTGTCGTGGAGTCGGCAATTGCACTGGATACGACAGCCCAAA	
hsa-miR-100-3p	GTCGTATCCAGTGCGTGTCGTGGAGTCGGCAATTGCACTGGATACGACCATACCTA	
hsa-miR-4286	GTCGTATCCAGTGCGTGTCGTGGAGTCGGCAATTGCACTGGATACGACGGTACCAG	
hsa-miR-582-5p	GTCGTATCCAGTGCGTGTCGTGGAGTCGGCAATTGCACTGGATACGACAGTAACTG	
hsa-miR-210-3p	GTCGTATCCAGTGCGTGTCGTGGAGTCGGCAATTGCACTGGATACGACTCAGCCGC	
hsa-miR-30e-3p	GTCGTATCCAGTGCGTGTCGTGGAGTCGGCAATTGCACTGGATACGACGCTGTAAA	
cel-miR-39	GTCGTATCCAGTGCGTGTCGTGGAGTCGGCAATTGCACTGGATACGACCAAGCT	

cel-miR-54	GTCGTATCCAGTGCGTGTCGTGGAGTCGGCAATTGCACTGGATACGACCTCGGA	
Stem-loop RT-PCR primers		
hsa-miR-25-3p	ACGTCTACACTCCATTGCACTTGTCTCGG	CAGTGCGTGTCGTGGAGT
hsa-miR-1-1-3p	ACGTGGAATGTAAAGAAGTATGTATGTC	CAGTGCGTGTCGTGGAGT
hsa-miR-145-5p	ATAGCAGTCCAGTTTTCCCAGGAA	CAGTGCGTGTCGTGGAGT
hsa-miR-4763-3p	TAACAAAGGCAGGGGCTGGTGCTGG	CAGTGCGTGTCGTGGAGT
hsa-miR-630	TCAGCGAGTATTCTGTACCAGGG	CAGTGCGTGTCGTGGAGT
hsa-miR-1229-5p	TATCATGTGGGTAGGGTTTGGGGGA	CAGTGCGTGTCGTGGAGT
hsa-miR-5703	ACTACCTACAGGAGAAGTCGGG	CAGTGCGTGTCGTGGAGT
hsa-miR-6727-5p	TATCATCTCGGGGCAGGCGGCTGG	CAGTGCGTGTCGTGGAGT
hsa-miR-328-5p	TATACTGGGGGGGCAGGAGGGGCT	CAGTGCGTGTCGTGGAGT
hsa-miR-5787	TATACTGGGCTGGGGCGCGGG	CAGTGCGTGTCGTGGAGT
hsa-miR-34a-3p	TCGGACCAATCAGCAAGTATACT	CAGTGCGTGTCGTGGAGT
hsa-miR-4516	ACTATCTACGGGAGAAGGGTC	CAGTGCGTGTCGTGGAGT
hsa-miR-4459	ATTCATCCAGGAGGCGGAGGAGG	CAGTGCGTGTCGTGGAGT
hsa-miR-6090	ATTCATATAGGGGAGCGAGGGGC	CAGTGCGTGTCGTGGAGT
hsa-miR-7107-5p	ACTAATTCGGCCTGGGGAGGAGG	CAGTGCGTGTCGTGGAGT
hsa-miR-6068	ATTGATCCTGCGAGTCTCCGGC	CAGTGCGTGTCGTGGAGT
hsa-miR-6869-5p	TAACATGTGAGTAGTGGCGCGCG	CAGTGCGTGTCGTGGAGT
hsa-miR-33a-5p	ACTCCGTACGTAGTGCATTGTAGTTGCA	CAGTGCGTGTCGTGGAGT
hsa-miR-582-3p	CTCGACTAACTGGTTGAACAACT	CAGTGCGTGTCGTGGAGT
hsa-miR-30c-2-3p	ACATCGCTGGGAGAAGGCTGTTT	CAGTGCGTGTCGTGGAGT
hsa-miR-181d-5p	TCTGACAACATTCATTGTTGTCGG	CAGTGCGTGTCGTGGAGT
hsa-miR-186-5p	GACTATCAAAGAATTCTCCTTTT	CAGTGCGTGTCGTGGAGT
hsa-miR-100-3p	TGCAGCCAAGCTTGTATCTATAG	CAGTGCGTGTCGTGGAGT
hsa-miR-4286	TCGACGTATCATGATACCCCACTCCTGG	CAGTGCGTGTCGTGGAGT
hsa-miR-582-5p	ACTGCATTACAGTTGTTCAACCAG	CAGTGCGTGTCGTGGAGT
hsa-miR-210-3p	CTAGAATAACATCTGTGCGTGTGACAGCG	CAGTGCGTGTCGTGGAGT
hsa-miR-30e-3p	TCACTGTACGTACTTTCAGTCGGATGTTT	CAGTGCGTGTCGTGGAGT
cel-miR-39	TCACCGGGTGTAAATCAGCTTGGTCGTA	CAGTGCGTGTCGTGGAGT
cel-miR-54	TACCCGTAATCTTCATAATCCGAGGTCG	CAGTGCGTGTCGTGGAGT
hsa-pre-miR-25	CCAGTGTTGAGAGGCGGAGAC	ATTGCCACTGTCAGACCGAGACAAGTGC
hsa-pre-miR-1-1	GAAACATACTTCTTTATATGCCCAT	ATTGCCCTATGTATGAAGAAATGTAAGGTAT
U6	CTCGCTTCGGCAGCACA	AACGCTTCACGAATTTGCGT

Supplementary Table 6. Sequences of siRNAs, primers and probes used in this study.

Sequences of siRNA	
siControl	5'-UUCUCCGAACGUGUCACGUTT-3'
METTL3-siRNA-#1	5'-GGAGAUCCUAGAGCUAUUATT-3'
METTL3-siRNA-#2	5'-GCACAUCCUACUCUUGUAATT-3'
NFIC-siRNA-#1	5'-CCACGAGUAGCAGCCGCAATT-3'
NFIC-siRNA-#2	5'-GCAACUGGACGGAGGACAUTT-3'
PHLPP2-siRNA-#1	5'-GACCUCUUCAGAUCGUUUATT-3'
PHLPP2-siRNA-#2	5'-CUUGGGUUGUUUCCUAUAUTT-3'
p70S6K-siRNA-#1	5'-GUGCCAAUCAGGUCUUUCUTT-3'
p70S6K-siRNA-#2	5'-GCACCUGCGUAUGAAUCUATT-3'
NKAP-siRNA-#1	5'-GGUCUAAGAAGAGCAGAAATT-3'
NKAP-siRNA-#2	5'-CCAGUGAUGAAGAUAACAATT-3'
miR-25-3p mimic	5'-CAUUGCACUUGUCUCGGUCUGA-3'
miR-25-3p inhibitor	5'-UCAGACCGAGACAAGUGCAAUG-3'
Primers for DNA methylation-specific PCR	
METTL3-methylated Forward	5'-GAGGGCGGATTACGAGGTGAGGAGT-3'
METTL3-methylated Reverse	5'-TCCTACCGAACCTCCCGAATAACT-3'
METTL3-unmethylated Forward	5'-GAGGGTGGATTATGAGGTGAGGAGT-3'
METTL3-unmethylated Reverse	5'- TCCTACCAAACCTCCCAAATAACT -3'
Primers used for ChIP-qPCR	
METTL3-CHIP-Forward	5'- TGTGGTGGCTCACGCCTGTAATCC-3'
METTL3-CHIP-Reverse	5'- GATCTCGGCTCACAGCAACCTCCAC-3'
Primers used for *in vitro* transcription	
Pri-miR-25-Forward	5'-ATTGCCTAATACGACTCACTATAGGGAGACACGTTCTCTCTGCCAATT-3'
Pri-miR-25-Reverse	5'-AAGAATTTGGGCCGGCACTGTCAGACCGA-3'
Pri-miR-1-1-Forward	5'-ATTGCCTAATACGACTCACTATAGGGAGAGGGCCCGGCAGAGA-3'
Pri-miR-1-1-Reverse	5'-CAATAACCCCGCCTCCCCCCCCCGCC-3'
Probes used for Northern blot	
Pri-miR-25	5'-UCAGCCAAGAAGACAAUUGGCAGA-3'
Pri-miR-1-1	5'-AACUUCUUCCCUGGAGUCUACUGC-3'
Pre-miR-25	5'-AATGCCCAGGGCAGCGTCCAG-3'
Pre-miR-1-1	5'-ATAGCTTAGCAGGTCCATAT-3'
miR-25-3p	5'-UCAGACCAGACAAGUGCAAUG-3'
Primers used for MeRIP-qPCR and CLIP-qPCR	
miR-25-Forward	5'- TAAGAAACTCCGGGACTGGCCAGTG -3'
miR-25-Reverse	5'- CACTGTCAGACCGAGACAAGTGCAA -3'
