# Supplementary material for: Excessive miR-25-3p maturation via N6-methyladenosine stimulated by cigarette smoke promotes pancreatic cancer progression
Source: Nat Commun. 2019 Apr 23;10:1858. doi: 10.1038/s41467-019-09712-x (PMC6478927; doi:10.1038/s41467-019-09712-x)
Supplement: Supplementary file 3 — Description of Additional Supplementary Files [file 41467_2019_9712_MOESM3_ESM.pdf]

Description of Additional Supplementary Files

File Name: Supplementary Data 1
Description: Characteristics of individuals with pancreatic ductal adenocarcinoma in this study.

File Name: Supplementary Data 2
Description: Mass spectrometry data for [A]pri-miR-25-interacting proteins.

File Name: Supplementary Data 3
Description: Mass spectrometry data for [m^6A]pri-miR-25-interacting proteins.

File Name: Supplementary Data 4
Description: Mass spectrometry data for DGCR8-interacting proteins.
