# Supplementary material for: Excessive miR-25-3p maturation via N6-methyladenosine stimulated by cigarette smoke promotes pancreatic cancer progression
Source: Nat Commun. 2019 Apr 23;10:1858. doi: 10.1038/s41467-019-09712-x (PMC6478927; doi:10.1038/s41467-019-09712-x)
Supplement: Supplementary file 8 — Reporting Summary [file 41467_2019_9712_MOESM8_ESM.pdf]

natureresearch

Corresponding author(s): Dongxin Lin

Last updated by author(s): Mar 6, 2019

Reporting Summary

Nature Research wishes to improve the reproducibility of the work that we publish. This form provides structure for consistency and transparency in reporting. For further information on Nature Research policies, see Authors & Referees and the Editorial Policy Checklist.

Statistics

For all statistical analyses, confirm that the following items are present in the figure legend, table legend, main text, or Methods section.

n/a	Confirmed	
☐	☒	The exact sample size (n) for each experimental group/condition, given as a discrete number and unit of measurement
☐	☒	A statement on whether measurements were taken from distinct samples or whether the same sample was measured repeatedly
☐	☒	The statistical test(s) used AND whether they are one- or two-sided<br>*Only common tests should be described solely by name; describe more complex techniques in the Methods section.*
☒	☐	A description of all covariates tested
☐	☒	A description of any assumptions or corrections, such as tests of normality and adjustment for multiple comparisons
☐	☒	A full description of the statistical parameters including central tendency (e.g. means) or other basic estimates (e.g. regression coefficient) AND variation (e.g. standard deviation) or associated estimates of uncertainty (e.g. confidence intervals)
☐	☒	For null hypothesis testing, the test statistic (e.g. F, t, r) with confidence intervals, effect sizes, degrees of freedom and P value noted<br>*Give P values as exact values whenever suitable.*
☒	☐	For Bayesian analysis, information on the choice of priors and Markov chain Monte Carlo settings
☒	☐	For hierarchical and complex designs, identification of the appropriate level for tests and full reporting of outcomes
☐	☒	Estimates of effect sizes (e.g. Cohen's d, Pearson's r), indicating how they were calculated

Our web collection on statistics for biologists contains articles on many of the points above.

Software and code

Policy information about availability of computer code

Data collection	Illumina HiSeq2500; Illumina HiSeq X Ten
Data analysis	Microsoft Excel 2016, ImageJ software, Photoshop (Adobe), GraphPad Prism 6 software, SPSS software (version 20.0; IBM SPSS), R software for statistical computing.

For manuscripts utilizing custom algorithms or software that are central to the research but not yet described in published literature, software must be made available to editors/reviewers. We strongly encourage code deposition in a community repository (e.g. GitHub). See the Nature Research guidelines for submitting code & software for further information.

Data

Policy information about availability of data

All manuscripts must include a data availability statement. This statement should provide the following information, where applicable:

- Accession codes, unique identifiers, or web links for publicly available datasets
- A list of figures that have associated raw data
- A description of any restrictions on data availability

Microarray data have been deposited at Gene Expression Omnibus (GSE103994); miCLIP and NKAP iCLIP data have been deposited at Gene Expression Omnibus (GSE116425).
The source data underlying Figs. 1, 2, 3, 4, 5, 6 and 7 and Supplementary Figs. 2, 3, 4, 5, and 7 are provided as Source Data files.
A reporting summary for this article is available as a Supplementary Information file.

October 2018

Field-specific reporting

Please select the one below that is the best fit for your research. If you are not sure, read the appropriate sections before making your selection.

☒ Life sciences ☐ Behavioural & social sciences ☐ Ecological, evolutionary & environmental sciences

For a reference copy of the document with all sections, see nature.com/documents/nr-reporting-summary-flat.pdf

Life sciences study design

All studies must disclose on these points even when the disclosure is negative.

Sample size	The sample size of the TCGA and GEO datasets was determined by the number of tumor samples analyzed with each technology. Sample size and number of independent experiments are always clearly stated in the figure legend or in the Methods section. Three to more independent results were used to perform statistical analyses. If less, no statistics were performed from these samples. All raw data required for statistical tests are indicated in supplementary source data.
Data exclusions	No data were excluded from analysis.
Replication	Experiments in the article were reliably reproduced, replication were described in the figure legends.
Randomization	Animals with similar age and weight were randomly allocated to experimental groups.
Blinding	Investigators were not blinded to group allocation during data collection and/or analysis.

Reporting for specific materials, systems and methods

We require information from authors about some types of materials, experimental systems and methods used in many studies. Here, indicate whether each material, system or method listed is relevant to your study. If you are not sure if a list item applies to your research, read the appropriate section before selecting a response.

Materials & experimental systems

n/a	Involved in the study	
☐	☒	Antibodies
☐	☒	Eukaryotic cell lines
☒	☐	Palaeontology
☐	☒	Animals and other organisms
☐	☒	Human research participants
☒	☐	Clinical data

Methods

n/a	Involved in the study	
☒	☐	ChIP-seq
☒	☐	Flow cytometry
☒	☐	MRI-based neuroimaging

Antibodies

Antibodies used

Rabbit anti-METTL3 antibody (WB: dil. 1:4000, Supplier: Abcam, Cat.: ab195352)
Rabbit anti-PHLPP1 antibody (WB: dil. 1:2000, Supplier: Abcam, Cat.: ab71277)
Rabbit anti-PHLPP2 antibody (WB: dil. 1:4000, Supplier: Abcam, Cat.: ab71973)
Rabbit anti-AKT1/2/3 antibody (WB: dil. 1:2000, Supplier: Abcam, Cat.: ab126811)
Rabbit anti-p-AKT1/2/3 (p-S472+S473+S474; WB: dil. 1:2000, Supplier: Abcam, Cat.: ab183758)
Rabbit anti-p70S6K antibody (WB: dil. 1:2000, Supplier: Abcam, Cat.: ab32359)
Rabbit anti-p-p70S6K antibody (p-T389; WB: dil. 1:2000, Supplier: Abcam, Cat.: ab126818)
Rabbit anti-NKAP antibody (WB: dil. 1:1000, Supplier: Abcam, Cat.: ab121121)
Rabbit anti-IF4A2 antibody (WB: dil. 1:2000, Supplier: Abcam, Cat.: ab31218)
Rabbit anti-SLU7 antibody (WB: dil. 1:2000, Supplier: Abcam, Cat.: ab151462)
Rabbit anti-PCMD1 antibody (WB: dil. 1:1000, Supplier: Abcam, Cat.: ab121858)
Rabbit anti-PLXA4 antibody (WB: dil. 1:2000, Supplier: Abcam, Cat.: ab127892)
Rabbit anti-CENPJ antibody (WB: dil. 1:1000, Supplier: Abcam, Cat.: ab26052)
Rabbit anti-FLIP1 antibody (WB: dil. 1:1000, Supplier: Abcam, Cat.: ab205925)
Rabbit anti-DROSHA antibody (WB: dil. 1:1000, Supplier: Abcam, Cat.: ab12286)
Rabbit anti-DGCR8 antibody (WB: dil. 1:2000; 5 ug for IP and RIP; Supplier: Abcam, Cat.: ab127892)
Mouse anti-NFIC antibody (WB: dil. 1:2000; 5 ug for IP, RIP and ChIP; Supplier: Santa Cruz Biotechnology, Cat.: sc-74444)
Mouse anti-FLAG tag antibody (WB: dil. 1:2000; 5 ug for IP and RIP, 15 ug for CLIP; Supplier: Sigma; Cat.: F1804)
Mouse anti-6×His tag antibody (WB: dil. 1:2000; 5 ug for IP; Supplier: Sigma; Cat.: SAB2702218)
Mouse anti-beta-ACTIN antibody (WB: dil. 1:10000, Supplier: Proteintech; Cat.: 66009-1-Ig)

Validation

When manufacturer validation was doubtful, antibodies were tested on positive controls and/or positive controls.

Eukaryotic cell lines

Policy information about cell lines

Cell line source(s)	Human PDAC cell lines PANC-1, BXPC-3 and SW1990 and embryonic kidney cells 293T were purchased from the Cell Bank of Type Culture Collection of the Chinese Academy of Sciences Shanghai Institute of Biochemistry and Cell Biology. Human immortalized pancreatic duct epithelial cell line HPDE6-C7 was purchased from Biotechnology Company.
Authentication	All cell lines are commercial and authenticated.
Mycoplasma contamination	All cell lines were tested to be mycoplasma negative.
Commonly misidentified lines (See ICLAC register)	No cell lines used in this study were found in the database of commonly misidentified cell lines that is maintained by ICLAC and NCBI Biosample.

Animals and other organisms

Policy information about studies involving animals; ARRIVE guidelines recommended for reporting animal research

Laboratory animals	Female BALB/c nude mice, aged 4–5 weeks, purchased from the Beijing Vital River Laboratory Animal Technology were used in this study.
Wild animals	No wild animals were used in this study.
Field-collected samples	No field-collected samples were used in this study.
Ethics oversight	All animal experiments were performed in accordance with relevant institutional and national guidelines and regulations.

Note that full information on the approval of the study protocol must also be provided in the manuscript.

Human research participants

Policy information about studies involving human research participants

Population characteristics	Surgically removed PDAC and paired non-tumor tissue samples were obtained from individuals who underwent pancreatectomy at Sun Yat-sen Memorial Hospital, Sun Yat-sen University (Guangzhou, China) and Cancer Hospital, Chinese Academy of Medical Sciences (Beijing, China) between 2010 and 2016. PDAC was diagnosed by histopathological examination.
Recruitment	Demography characteristics and clinical information of individuals were obtained from medical records. All the individuals underwent pancreatectomy received no chemotherapy or radiotherapy before surgery. The bio-specimens from each individual were collected at the time of surgery.
Ethics oversight	This study was approved by the Institutional Review Board of the Sun Yat-sen Memorial Hospital and Chinese Academy of Medical Sciences, Cancer Hospital.

Note that full information on the approval of the study protocol must also be provided in the manuscript.
